# Harnessing Augmented Reality for Increasing the Awareness of Food Waste Amongst Dutch Consumers

**DOI:** 10.1007/s41133-022-00057-7

**Published:** 2022-07-01

**Authors:** Dolf Honee, William Hurst, Antonius Johannus Luttikhold

**Affiliations:** 1 Information Technology Group, Wageningen University and Research, Wageningen, The Netherlands; 2 Wastewatchers, Utrecht, The Netherlands

**Keywords:** Augmented reality, Food waste, Catering industry, Consumer

## Abstract

Food waste is a significant challenge, and our societal behaviours play a role in the amount of food items discarded. Thus, an effective method to inform consumers about high wastage patterns may help reduce the amount thrown away. This research investigates how Augmented Reality can be harnessed to enlighten consumers and work towards addressing high food waste patterns. Yet research on this topic is still very much in its infancy. To pursue this solution, food behaviour data are employed to provide an insight into how much is wasted from 9 catering industry locations in the Netherlands. An Augmented Reality application is developed, where models of food are projected onto real-world environments to provide scale on waste over a 7-day period. A quantitative evaluation of higher-education attendees demonstrated the approach has potential to incentivise reduction in waste.

**Supplementary Information:**

The online version contains supplementary material available at 10.1007/s41133-022-00057-7.

## Introduction

Food waste is a significant problem in the current economy. A considerable volume of food is thrown away or wasted. In the Netherlands, about 9.5% of all bought food is wasted yearly; this equates to 34.3 kg of solid foods [1] and equivalent to 154 kg carbon dioxide (CO_2_) per person per year. Furthermore, this accounts for 8–10% of all the greenhouse gasses emitted by the Netherlands [1]. In wider Europe, about 186 Mt CO_2_-eq is attributed to food waste [2]. This has resulted in food campaigns, such as the *clean dish, clean conscience!* initiative in Portugal, to help increase awareness for food waste behaviours [3]. However, in general, as confirmed by UN Sustainable Development Goal SDG 12.3, more solutions are required to address the pattern of waste.

Understanding the volume of waste is a particular challenge for consumers, and digital technologies, such as Augmented Reality (AR), could provide a supportive metric to increase awareness with the potential to inspire consumers positively [4] by providing a visual aid to understand the amount of waste produced. AR is a suitable technology for this purpose, as it is becoming more accessible and widespread, largely due to the capabilities of smart phones. Because of this, in the current environment, almost every consumer has an AR-capable device, making the technology an ideal solution for visual communication. For instance, each smart phone is equipped with a screen, camera and hardware, making the devices suitable for running modern-day AR applications. Furthermore, AR is already used regularly in education and communicative applications. For example, according to [5], AR is a suitable method to deliver educational material due to its ability to deliver a learning experience that combines experiential, kinaesthetic and didactic learning. Similarly, in a study by Nicholson-Cole et al. [6], visual communication showed a positive impact on the perceived importance of the prominent subject of climate change. Thus, demonstrating that users are becoming familiar with AR applications and that a visual element is an ideal metric for communication.

It may be challenging to grasp what it means to waste 8–10% of bought food, but a visual representation of this may have a clearer impact. The theory is that, through the use of an AR experience, consumers may be more inclined to both understand and work towards a reduction of food waste behaviours. In order to address this, the study investigates suitable AR approaches (from related works) for the creation a food waste application. An in-depth analysis of related work is conducted using a snowballing technique, in which only open-access articles are considered for repeatability of the investigation. Based on the findings, a prototype AR application for food waste visualisation is designed and developed using the Aryzon AR Software Development Kit (SDK). At the time of writing this article, Aryzon is an emerging technology and yet to be employed within the food waste visualisation domain. Therefore, the technology is not found within the related works discussion but is an ideal solution due to the integration with Unity game engine and that it caters for both screen-based and wearable AR deployment. Evaluation of the AR application involved participants attending a higher-education institute, a similar approach to the work by Pinto et al. in [2] for the promotion of food waste reduction. However, access to this participant group was limited due to social distancing measures at the time of testing.

The remainder of the article is as follows. “Related Work” section provides a background discussion and literature review of the current use of AR for food waste applications (at the time of writing this, there are relatively few articles on this topic). “Materials and Methods” section discusses the methodology adopted for the development of a AR application, and “Results” section provides and overview of the implementation. The article findings are discussed in “Discussion” section, and conclusions are presented in “Conclusion” section.

## Related Work

AR has a broad scope of applications, examples include teaching chemistry in higher education [7], support for spinal surgery [8] and assisting with building tunnels in construction [9], to name but a few. As discussed, the focus of this article is on AR in the food waste domain, which is a research concept that is very much in its infancy. Therefore, in order to design and develop an effective AR solution, first an investigation is required into related works in order to ascertain the appropriate features to employ and obstacles to avoid during the design and implementation of the solution. In this section, an overview of the field of interest is provided.

### Search Strategy

The focus of the related work is on two digital libraries IEEE Xplore and Science Direct. As AR is an emerging technology with continuous developments, only literature from the last five years (2016–2021) is considered in this study. A survey of the literature landscape demonstrated that a significant amount of AR-based articles are conference based. Therefore, the targeted sources include journals, conferences and workshops. For both libraries, a focused general search query is used to identify papers specifically addressing food waste and AR. The search catered for possible variations in the key words. This resulted in the following generalised search query, where 458 papers were identified (75 from Science Direct and 383 from IEEE Xplore).

1 $$ \left( {Augmented \, {{Re}} ality\;OR \, Mixed \, {{Re}} ality \, OR \, {{Im}} mersive \, OR \, Virtual \, OR \, 3D \, or \, XR} \right)\;AND\;\left( {Food \, waste\;OR\;Food \, loss} \right) $$

(1) is an abstracted example of the search string, as variations of Augmented and Mixed reality (e.g. AR, XR, MR) were included in the full search. Initially, the search string had a broad scope to ensure no potentially relevant research articles are missed. From the number of papers, the most relevant ones were filtered using the selection criteria presented in Table 1. The selection criteria are applied manually by reading the title and abstract of the studies. This reduced down the number of articles down to 15 (a full list is presented in Table 2). An immediate observation is that there are few articles available that combine both AR and food waste, demonstrating that the work presented in the article is within a potential research niche. In the following subsection, the findings from these 15 papers are discussed.

**Table 1:** Study selection criteria

No	Criterion
SC1	Papers without full text available
SC2	Papers not written in English
SC3	Duplicate publication from multiple sources
SC4	Papers do not relate to AR
SC5	Papers are not directly related to this study (i.e., not applicable to an AR app for food waste)

**Table 2:** Study selection criteria

Fuchs et al. [12]	2020	Li et al. [13]	2020
Fujimoto [14]	2018	Marto et al. [10]	2020
Gong et al. [15]	2021	Qiao et al. [16]	2019
Guillen et al. [17]	2021	Rodrigues et al. [11]	2019
Hoppenstedt et al. [18]	2019	Shea et al. [19]	2017
Ibáñez et al. [20]	2016	Song et al., [21]	2020
Kim et al. [22]	2021	Zhou et al. [9]	2020
Koutitas et al. [23]	2020		

### Findings

**Fig. 1:**
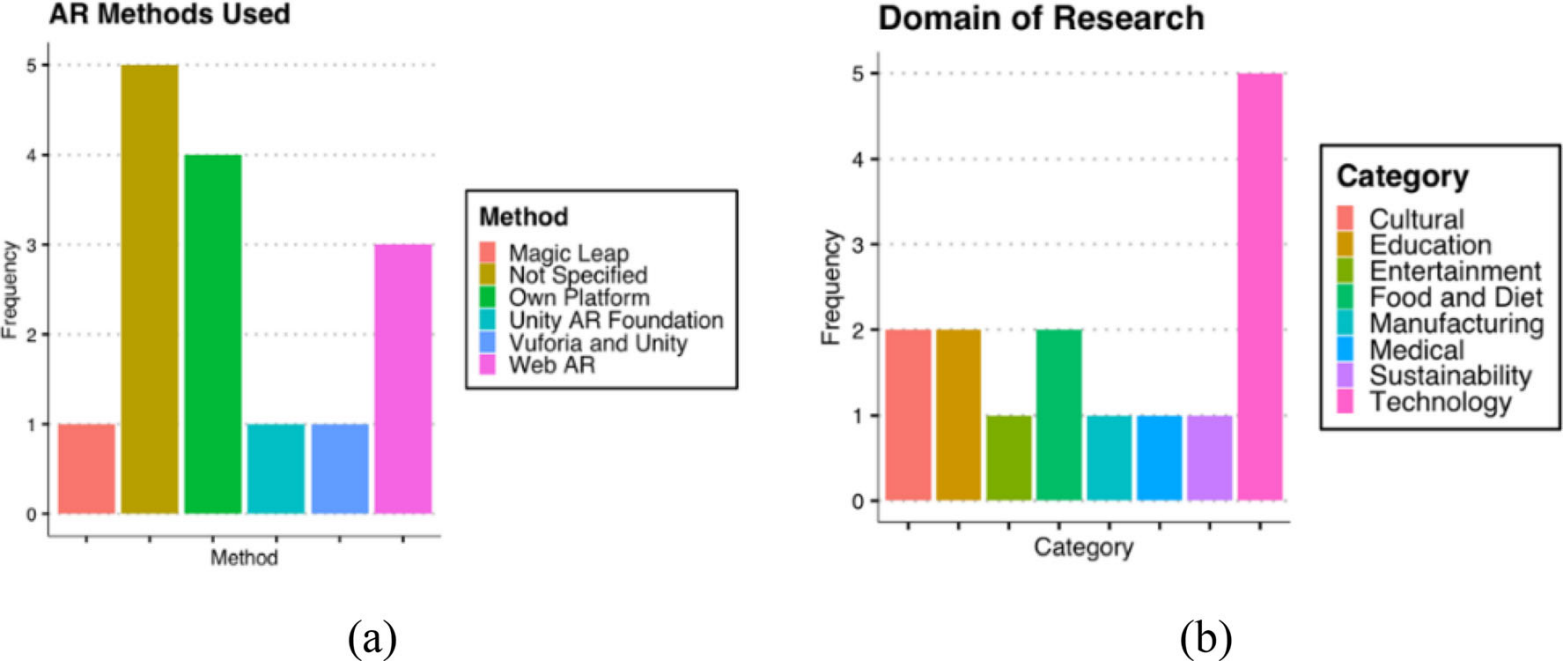
**a** AR methods used **b** Research Domains

Within the 15 papers, differing development platforms are employed for the construction of the AR applications. However, 5 studies did not state which platform they used (mostly because the investigations within the 5 articles are applied to the general implementation of AR). The most used platforms are self-created; an approach adopted by 4/15 articles, for example, the SensiMAR prototype by Marto et al., [10] and the M5SAR by Rodrigues et al. [11]. This customised approach is a suitable solution to work with, as the tools are specifically made for their intended goal. As Marto et al. [10] discuss, the use of a custom-built AR technology is necessary because no comparable technologies were available with the same features. However, a limitation of this method is that it has significant development costs due to the high levels of customisation. 3/15 studies used Web AR as their main technology for creating an application. Web AR is a flexible approach, as no app has to be installed and is compatible with almost any device that is AR-capable. A disadvantage of this method is that, at the time of this investigation, Web AR does not have a broad range features compared to other custom-built AR platforms, such as AR Core. Other platforms that are used in the articles include Unity AR, Vuforia, and Magic Leap, as displayed in Fig. 1a. The application domains of the 15 papers can be grouped into 8 categories, cultural, education, entertainment, food and diet, manufacturing, medical, sustainability and technology, as in Fig. 1b.

The range of the categories indicates how flexible AR is for deployment in varied settings. Table 3 displays the full findings and identified obstacles from the selected studies. Crucially, of the 15 papers analysed, none is in the domain of food waste specifically, but there were some synergies; for example, Fujimoto et al. [14] discuss the perceived deliciousness of food and how this can be increased by chroma and colour modifications. This would be in line with the findings in Marto et al. [10] where a demonstration is provided that additional sensory stimuli may impact the experience of a user within AR.

**Table 3:** Related work summary

Study ID	Findings	Obstacles	AR Methods	Research Domain	Features
Fuchs et al	Mixed reality (XR) can support healthier food choices	XR interventions are a promising field of research	Unity AR foundation	Food and Diet	Wearable device + App
Fujimoto et al	Digital representation of food may relate to its perceived deliciousness	Limited number of food types considered in the investigation	Web AR	Food and Diet	No wearable device, no app
Gong et al	Virtual reality (VR) may have a suitable user acceptance with the appropriate framework	Application depends heavily on user acceptance of the VR hardware	Vuforia and Unity	Manufacturing	Wearable device + App
Guillen et al	Gamification may offer user engagement benefits within sustainability subjects	There is a fine line between gamification and game-based activities – and the long-term impact is inconclusive	Not specified	Sustainability	No wearable device + App
Hoppenstedt et al	AR analytics may be a suitable replacement for 2D data– A wearable headset may improve user engagement	A wearable headset may be a possible stressor	Not specified	Medical	Wearable device + App
Ibáñez et al	AR can support with learning and information retention in education	Further study is necessary	Own platform	Education	Wearable device + App
Kim et al	User perception in AR may depend on interaction between virtual and physical	Perceived immersion may be disrupted when virtual objects overlap with physical objects	Magic leap	Technology	Wearable device + App
Koutitas et al	Reduce both computational resources and the induced network traffic, by caching images	For best performance, caching algorithm requires detailed information about the user and object locations	Not specified	Technology	Wearable device + App
Li et al	Web-based mobile AR and VR loading times and experience can be improved through on demand loading of 3D models	The number of animation tracks and the size of the models affect the optimisation of this method	Web AR	Technology	No wearable device—No app
Marto et al	Combination of sensory stimuli (audio, visual and smell) may improve enjoyment and knowledge retention in AR	Impact of sensory stimuli on overall experience changes widely combined with other stimuli	Own platform	Cultural	Wearable device + App
Qiao et al	Web AR has a promising future for mobile AR	Depends highly on internet speeds -Web based AR is still in its infancy stage	Web AR	Technology	No wearable device—No app
Rodrigues et al	Social influence, effort expectancy, and facilitating conditions are important in user acceptation in AR technology	Research only limited to one type of AR technology	Own platform	Cultural	Wearable device + App
Shea et al	Smartphones and their pervasive sensing possibilities can result in successful AR applications	AR apps with heavy pervasive sensing consumes significant battery power	Own platform	Entertainment	No wearable device + App
Song & Qiu	AR teaching methods may improve the learning progress of contemporary college students	Only investigated in political and ideological education – Not a replacement for conventional education	Not specified	Education	No wearable device + App
Zhou et al	Different display technologies may affect users’ visual comfort, interaction experience, learning experience, and outcome in hands-on learning	In terms of more complex learning activities and long-term learning further study needs to be done	Not specified	Technology	Wearable device + App

In terms of engagement, Hoppenstedt et al. [18] discuss that immersive analytics by means of AR are a suitable replacement for 2D representations of data and may be even better for retention. Furthermore, Fuchs et al. [12] highlight how the visual element of AR has an impact when users make a choice about which food they will buy. For the design of any future food waste applications, this may suggest altering the 3D models with chroma and partial-colour modifications to help make the models look more appetising and incentivise users to waste less of these foods. Therefore, in the application detailed in “Materials and Methods” section, the data will be presented through a combination of 3D models and 2D textual elements that further elaborate on the 3D models. In this way, the user is engaged by the stereoscopic images, where specific information (that would be difficult to represent in a 3D model) is provided through text overlay.

Regarding the hardware, one item of note is that a wearable device may be a possible stressor for users and this should be factored in when developing an AR application for food waste awareness. The evaluation process in this article takes note of this, by offering a testing process that makes use of both a screen and headset, allowing the user to choose. This approach also aligns with 9/15 papers, which investigated an application and a wearable headset combination, whereas 3/15 made use of only an application and no wearable headset, and another 3/15 made use of no installed application and no headset. Yet, according to Fuchs et al. (2020), headsets help to achieve high presence and immersions for the users. Moreover, a wearable headset may improve user engagement [18]. An installed application has the advantage that it can have 3D models pre-installed and allow for more complex features (an example of this can be the case of Pokémon Go! [19]).

At the time of writing this article, no AR technologies within the articles evaluated have been developed specifically for food waste. Based on other approaches in different categories, the findings indicate that the most suitable options for the design of an AR app for food waste would be to create an app using a custom-built SDK (e.g. Aryzon). This would cater for flexibility, both for the design and implementation process, as much of the framework and code libraries are already laid out.

## Materials and Methods

The design for an AR application for visualising food waste is put forward in this section. The approach is based on the findings from the literature review in “Related Work” section. Specifically, the design framework and data used for the application are presented. The section is concluded with an overview of the hardware and 3D construction process.

### Design

**Fig. 2:**
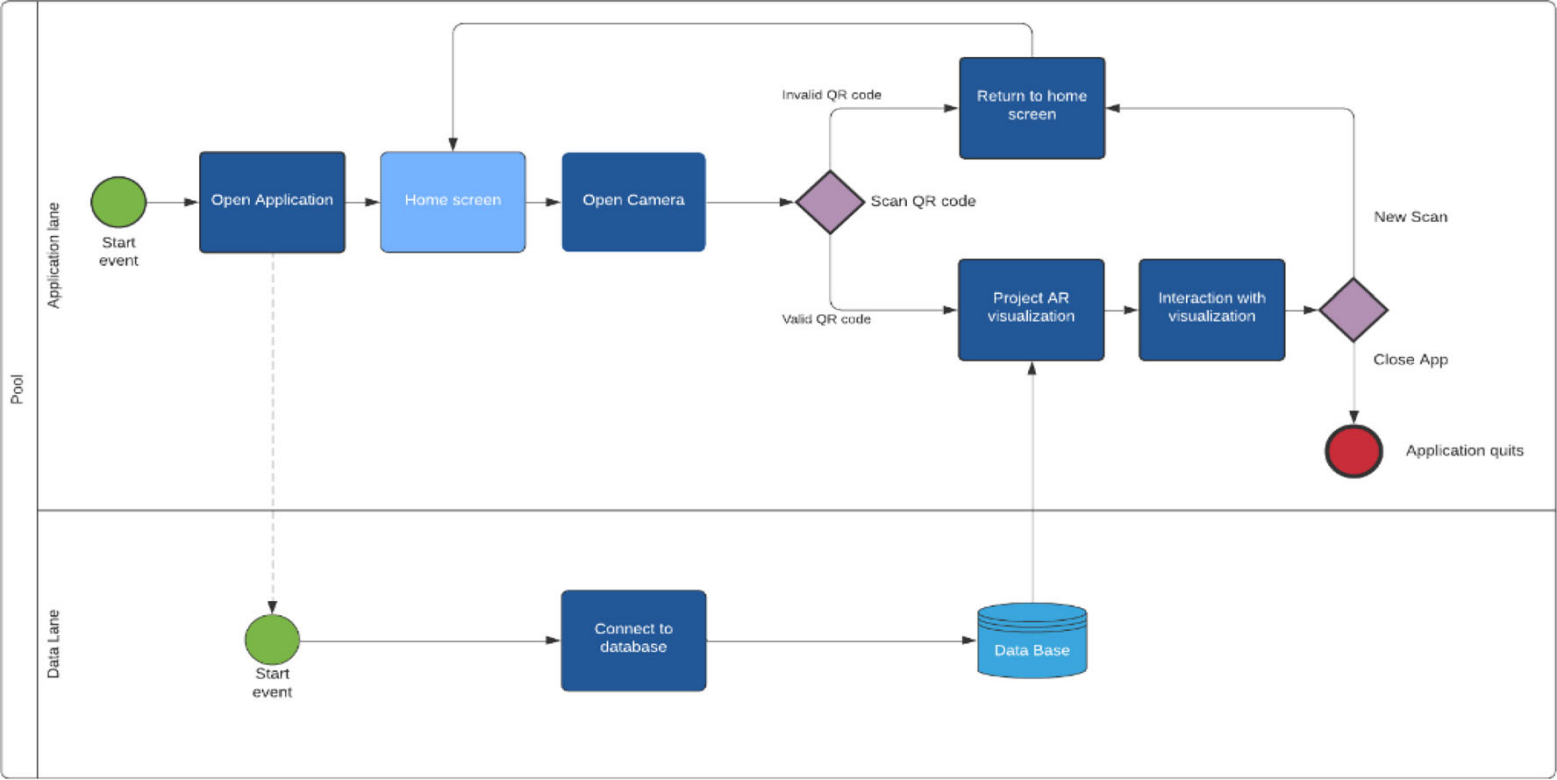
Framework Diagram for the AR application

A business process model and notation diagram is created below in Fig. 2 to represent how the app will function. The Aryzon SDK and the Unity platform are selected for the development of the application. Unity allows for custom creation within the application, whereas the Aryzon SDK supports the deployment process. In Fig. 2, when the user opens the application, they land on the home screen of the application. From here, users have the option to scan a QR code (if necessary) and open the camera or close the application. A QR code is the most suitable approach for interaction in a dynamic environment, such as a kitchen. Other approaches include using marker-less, which is the approach adopted for the trial in this article. This is because the Aryzon technology is able to track flat surfaces with a suitable accuracy for the evaluation. When a valid QR code is present or the user taps to a location on the screen, the application will gather the required data from an internal database and start to project the 3D visualizations in AR. When the user is finished, they have the option to close the app or start a new QR code scan.

### Data

To ensure that the application and visualisation produced are based on a real-world scenario, a dataset containing 40,000 entrees from nine different locations in the Netherlands from 01 August 2020 until 29 March 2021–2020 is employed (due to the signed agreement with the data use, the locations cannot be documented in the article to maintain privacy).

Within the data, the location, date, price and wasted item by category are detailed. Artefacts (measurement errors) were filtered from the raw data through consultation with the data provider. An example of the data can be seen below in Table 4 (where labels A, B and C are employed to maintain the anonymity of the locations).

**Table 4:** Example data

Date	Location ID	Product ID	Amount Produced	Amount Wasted	Price (Euros)	Day	Category
29–03-2021	A	278	1	0	1,72	1	Snacks
29–03-2021	A	531	2	1	1,15	1	Snacks
26–03-2021	A	927	6	0	5,31	5	Hot meal
26–03-2021	B	277	38	5	2,03	5	Snacks
26–03-2021	C	2349	8	7	0,91	5	Dairy

**Fig. 3:**
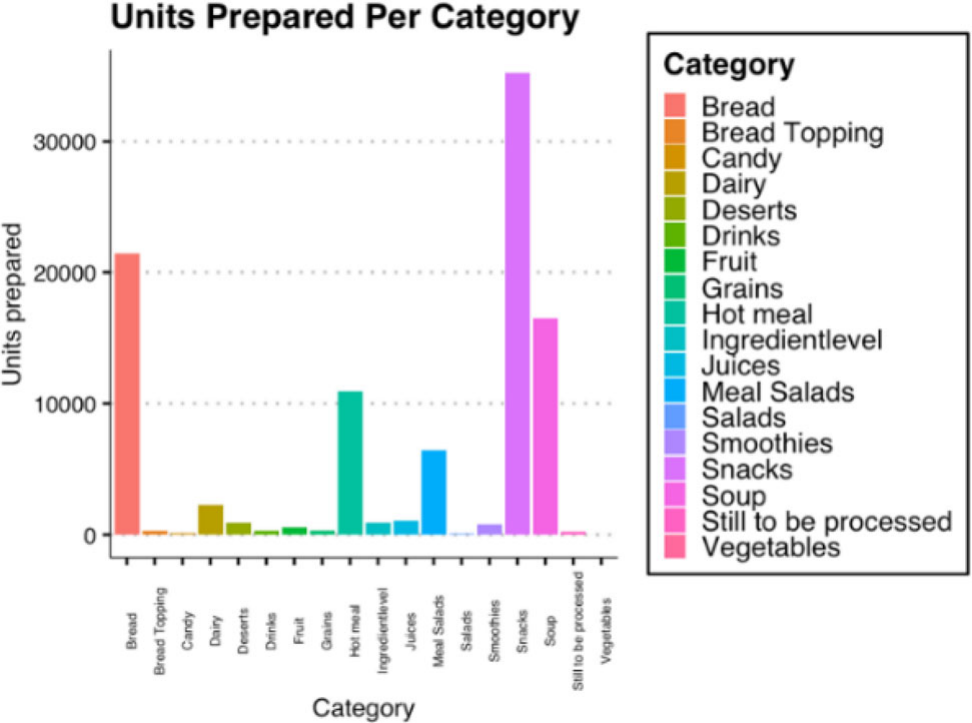
Food waste by category for 9 locations

When comparing waste measurements within the entire dataset, as in Fig. 3, bread and snacks are visibly the highest amongst the categories of left-over waste in all locations. However, bread and snacks are also the most sold categories.

Therefore, the percentage wasted compared to the units (sales) prepared by the cafeterias is factored in. The relative waste is calculated by dividing the total units (sales) wasted per category by the total units prepared per category. The formula is used to calculate this, as follows in (2):

2 $$ Lative\, waste\,per\,prepared\,unit = \frac{Total\,units\,of\,waste}{{Total\,units\,prepared}}*100\% $$

**Fig. 4:**
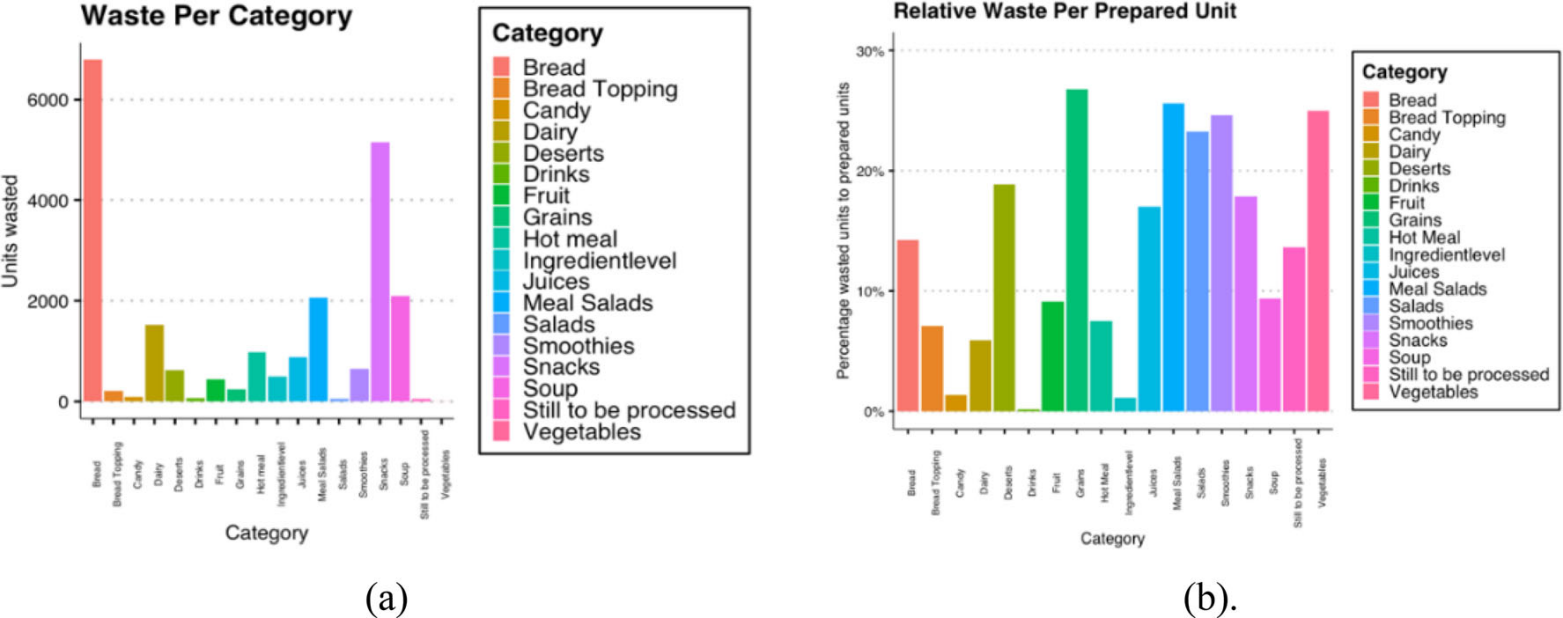
**a** Food waste per category. August 2020 – March 2021 **b** Relative waste per prepared unit per category. August 2020 – March 2021

It becomes clear that bread and snacks are not the largest producers of waste compared to their amounts produced. This is evident in Fig. 4a and b, where, with almost 30%, grains are the highest waste producer relative to units prepared.

For example, relatively fewer grains are purchased by the cafes and, thus, the total waste is not as significant as, for example, bread or soup. Waste data are also split into categories per days of the week. Tuesdays, Wednesdays, Thursdays and Fridays are very similar in their waste distribution.

**Fig. 5:**
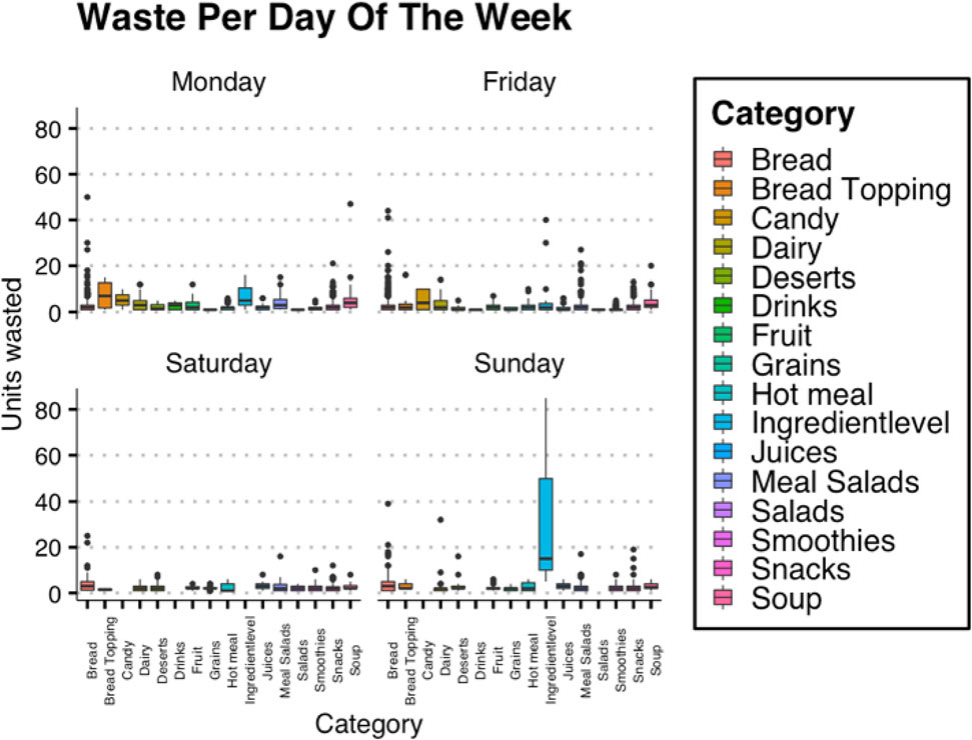
Boxplot of waste by category for 9 locations

In Fig. 5, boxplots are used to compare waste distribution between days of the week, with food category on the x-axis and units wasted on the y-axis. A difference is evident between the first day of the week, last day of the week and the weekend. On Friday, the amount of meal salads wasted is often higher. Monday sees more waste in the soup category. On Sunday, it is very apparent that the category Ingredient Level is wasted more compared to other days of the week.

### Visualisation

**Fig. 6:**
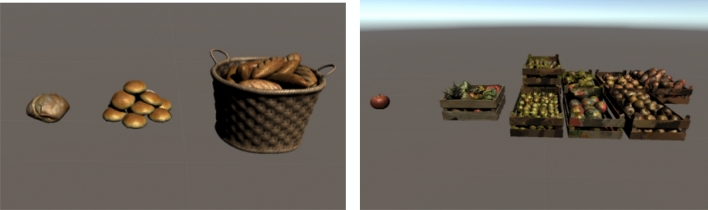
First trials with 3D models in unity

In order to represent this information within an AR application, the visualisation will be based on 3D models rendered in the Unity engine, where a prototype is developed with the Aryzon AR Studio SDK. The AR tracking from Aryzon in the application is able to recognise flat surfaces, without any major loading interruptions or errors. To minimise processing power demand, instead of rendering each amount separately, waste amounts will be categorized in 3D models that represent different amounts. For the Bread category for example, this means there will be a model for 1 unit of waste (e.g. one loaf of bread, or one snack item etc.), a model for 15 units of waste and a model for 25 units of waste. Conceptual examples are given for bread, drinks and fruits and vegetables in Table 5, and displayed in Unity in Fig. 6. Whilst drinks are include for the conceptualisation, only food is involved in the final application prototype.

**Table 5:** Waste units represented by 3D models

Amount (units)	3D Model (Bread)	3D Model (Drinks)	3D Model (Fruits & Vegetables)
1			
15			
25

A clear visualisation is the key feature of the application. A user should be able to identify what the visualisation represents and have an idea how much it is. It is, therefore, significant to make sure the 3D models are projected in realistic dimensions. Along with the models, additional data (in the form of text) are provided to support the visualisations. To recreate an appropriate level of realism, all the models have realistic textures that resemble the real-world equivalent.

### Hardware

**Fig. 7:**
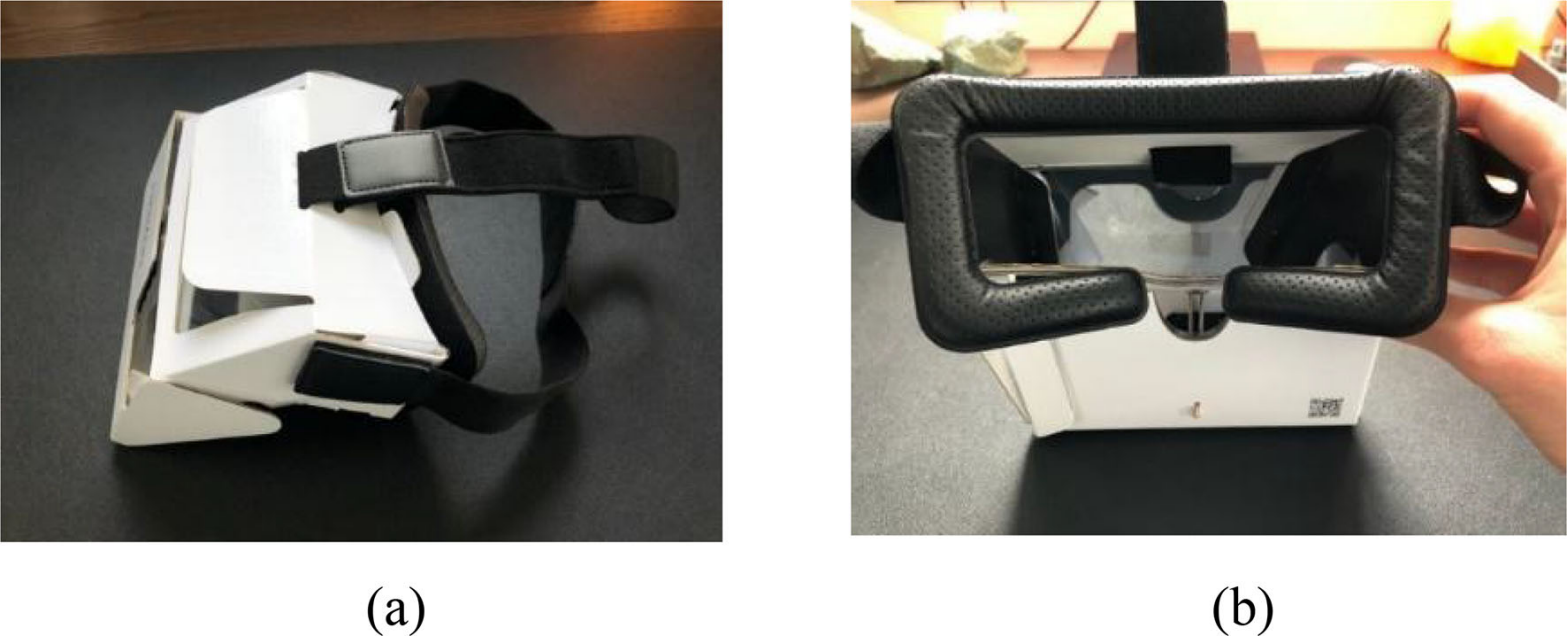
Aryzon’s cardboard headset **a** Side view **b** Front view into visor

For the application to work, an AR compatible phone is employed. This means for IOS devices running IOS 11 or later and for Android devices running Android 7.0 or later. Furthermore, the application is developed for use both with and without the Aryzon headset as displayed in Fig. 7.

The Aryzon headset is constructed from cardboard, which houses a mobile phone below the eye-level. Projections are created using an optical see-through approach, where mirrors combine 3D model with views of the physical world. This means the real-world image is as natural as possible. The headset has a head strap for a hands-free experience.

## Results

**Fig. 8:**
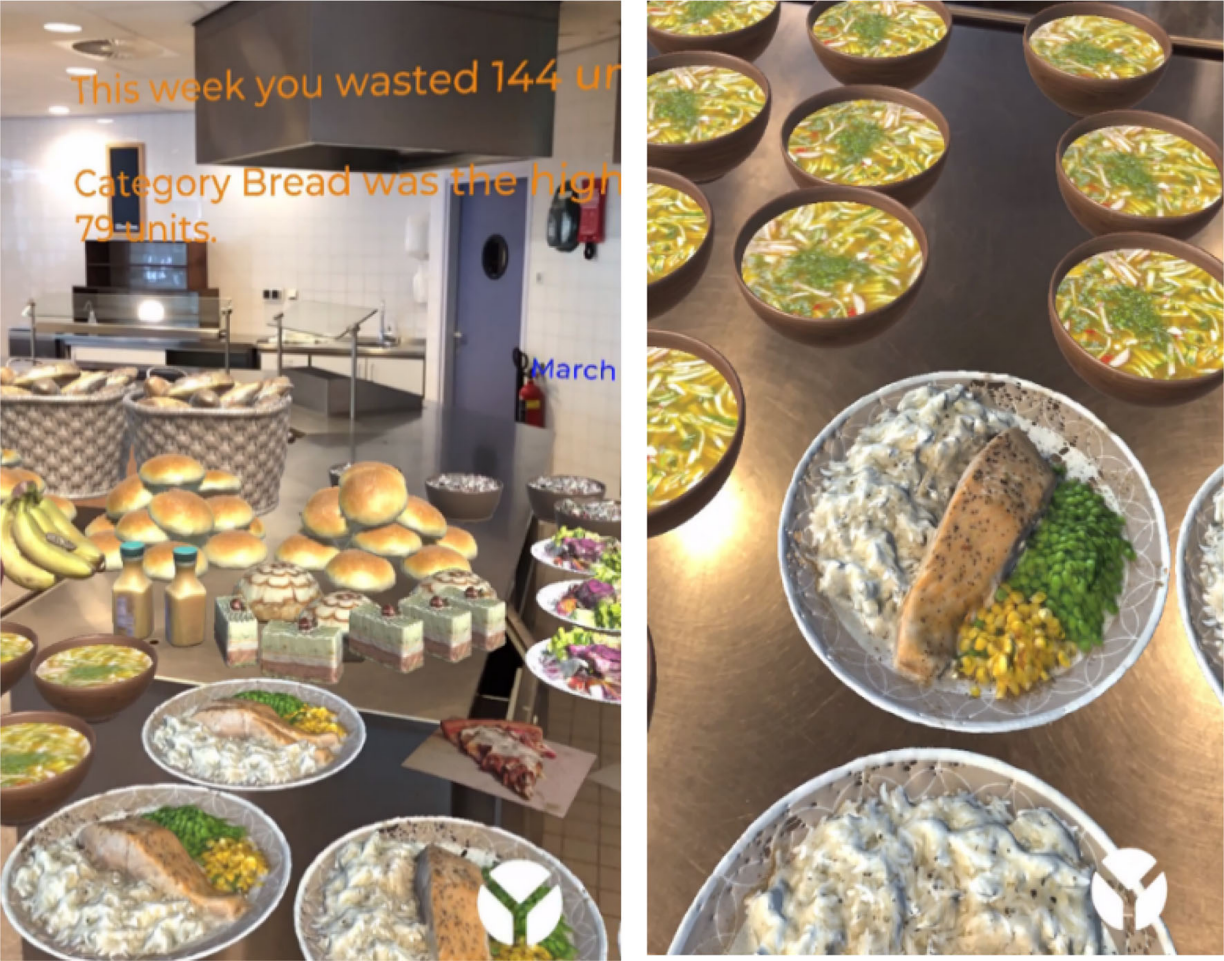
AR application trial

Testing is performed in the kitchen of the Leeuwenborch building from Wageningen University campus to evaluate the real-world application (Fig. 8). 25 testers (students of higher education) were randomly approached on premise to try out the application. The process involved asking users to use the AR application, view the 3D objects and read the text through means of viewing the application on a mobile phone screen. Out of these 25, 19 were available (access to a larger survey base was severely limited by the Covid-19 restrictions). 19 users also tried the AR application by using the headset for comparison with the screen-based approach. Users were then asked to complete 23 questions, 19 quantitative involving use of the Likert scale and 4 qualitative where text could be inserted (an overview of the questionnaire is provided in Online Appendix A).

The participants of this test were characterized as follows: 19 testers, all of them experts with mobile phones and with more than three years of background experience with smartphones and applications (aged between 18 and 24 years with, at least, a Bachelor’s Degree). Due to timeframe of the research and the current status of Covid-19, the test group was limited in size and diversity. In the tests, users could install the Android Package (APK) on their Android smartphone or try the application out the IOS smartphone provided by the authors. Users were introduced to the topic and after the demonstration were explained what the visualizations represented. The visualisation provided for the evaluation only concerned food waste and not drink. The inclusion of drink will be considered in future testing.

### Features

**Fig. 9:**
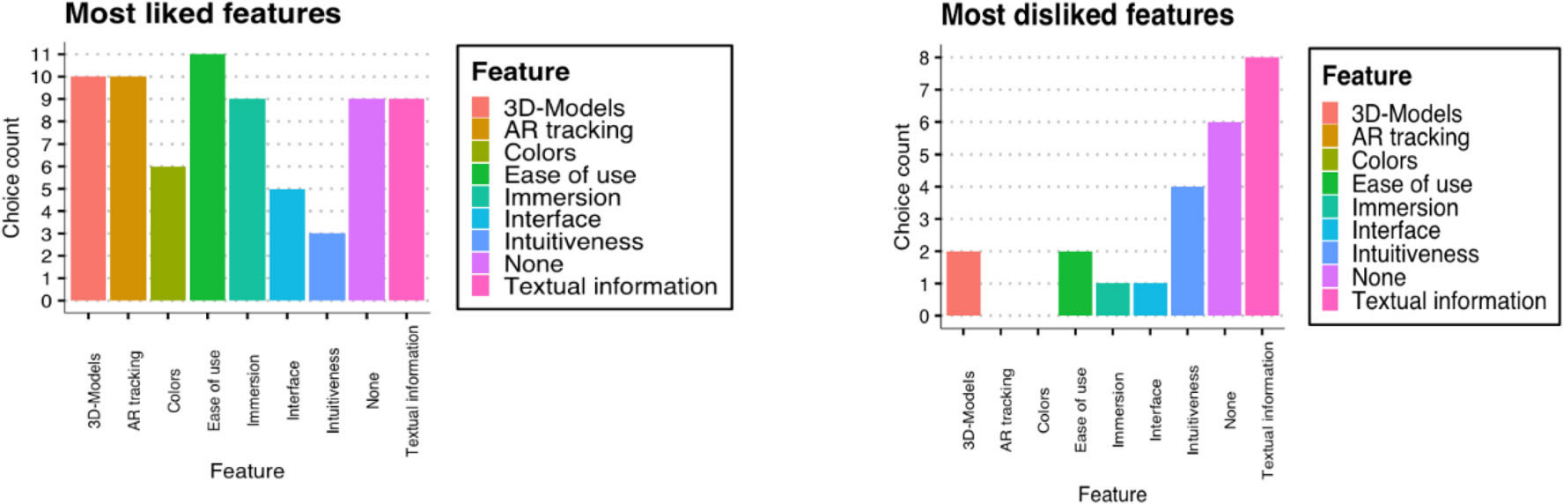
Most liked/disliked features

When asked about features, participants got the choice to select every feature they favoured the most; no limit was set to the number they are able to select (Fig. 9). 21% of participants favoured the ease of use of the application the most. This may be a positive indication for the implementation of the application within the catering business, as business professionals often may not want to spend time learning a new application that keeps them from their work. Second, with 18%, both the 3D models and the AR tracking were popular features. Overall, the most liked features were fairly widespread; where one apparent observation was that the intuitiveness was chosen as the least as a favourite feature. When asked what users liked the least, 33% said the textual information (Fig. 8). This may be a point to improve upon in further iterations of the application. It is also notable that 25% of the survey participants chose ‘none’ as their most disliked feature.

One user suggested to add CO_2_ emission information and another user suggested to add a menu. With regards to the immersion, all users felt immersed in the visualisation but all mentioned that the immersion can be improved upon. A further suggestion was to increase the polygon count on the 3D models (which would add more realism to the models) and another suggestion was to use more models in the same art style (e.g. to include drinks). The overall experience of the app was rated a 4/5 by the testers. The experience with the headset was the lowest with 3.4 out of 5 points.

### Food Waste

**Fig. 10:**
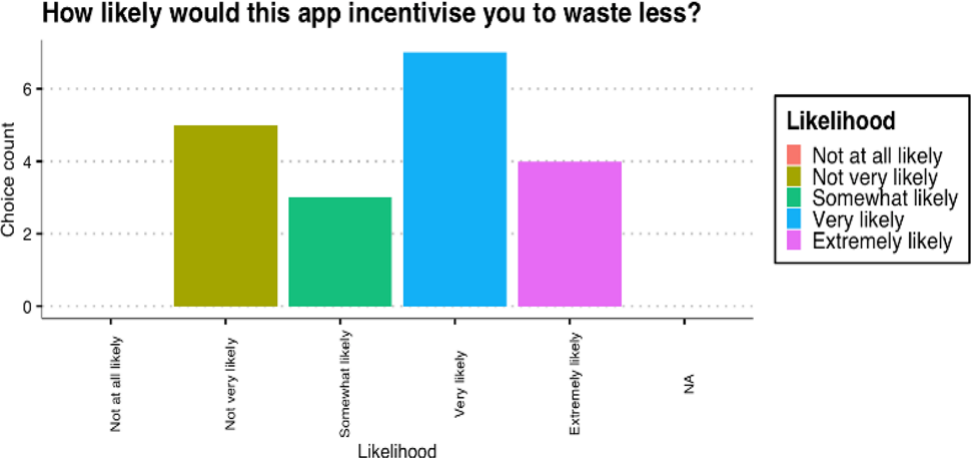
Likert scale on incentivisation to reduce waste

When asked about this application incentivising for reducing food waste, 21% of the participants gave it a 5/5 on a Likert scale and 37% gave the incentivization a 4/5. About 16% rated this with a 3/5. 26% thought the application was not very likely to have an impact. However, no users filled in the 1/5 option (Fig. 10). Notably, 60% of the users were surprised by the information the app gave and agreed that the application helped with understanding the level of waste. All participants agreed that the app clearly visualises which categories are wasted the most.

### Data Visualisation

**Fig. 11:**
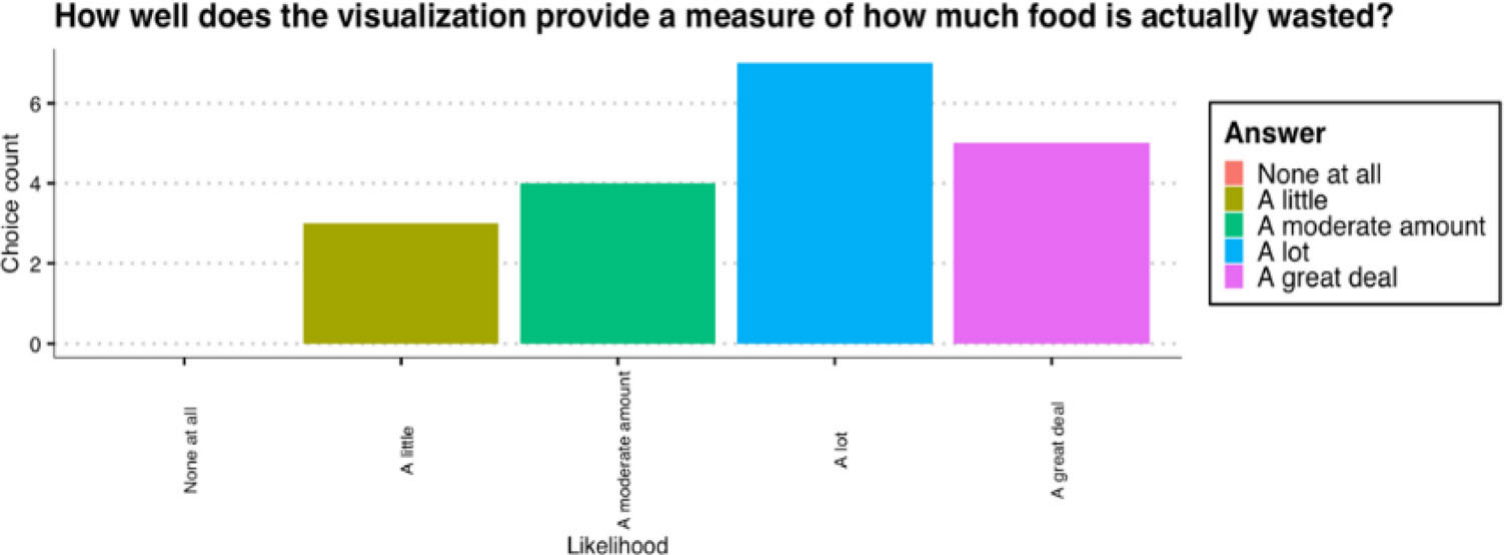
Measure of food waste provided

In terms of the usability to view the data, 42% would rather use the application without the headset. 26% would like to use the application with the headset and about 32% would prefer a hybrid approach (both with and without the headset), as displayed in Fig. 11.

Regarding the question as to whether the visualization provides a measure of how much food is wasted resulted in varied feedback. The most users (37%) gave this a 4/5 on the Likert scale. The rest of the answers were divided between 2/5, 3/5 and 5/5. However, all participants indicated that they were surprised by the amount wasted and answered that the waste was more than they expected. The realism of the models was rated a 4/5, with some users indicating that the realism can be improved to improve the overall immersion. Other suggestions for improvements in the app were more diversification between food categories and a clearer representation of the text. Compared to other methods of data visualisation, all users preferred the 3D visualisation of the application. Particularly for textual reports and static images, this method for visualisation of data may be preferred by about 70% of the testers.

## Discussion

As discussed, food waste is a significant issue in society, and prominent particularly within the current catering industry; demonstrated by the data overview in “Materials and Methods” section. Although 2D graphical representations may give an indication about how much food waste there is, a 3D representation would help with provide a level of scope. AR has a broad scope of uses, but relatively few apps exist in the domain of food waste. In fact, to the best of our knowledge, at the time of writing this article no applications specifically for AR with food waste exist. Yet, AR applications in other domains have shown to help with information retention and engagement, particularly in education.

From the literature review, it became clear that the research in food waste and AR is still in its infancy. Most domains of the researched AR papers are within the technology category, with 33%. 60% of papers made use of both a wearable device and an application. 27% of papers made use of their own platform and 33% of the papers did not specify which AR platform they used. The rest of the researched papers all used different platforms. One item of note is that a wearable device may be a possible stressor for users.

A variety of platforms also exist for building an AR application, each comes with their own advantages and disadvantages. The most suitable option for this research seemed to be to create an app with a wearable headset and a custom-built platform. However, the use of an existing SDK (e.g. Aryzon) caters for some flexibility with designing, as well as a relative quick development because the framework is already laid out.

Due to limited applications known about both subjects, this research is an opportunity to learn more about how AR can influence food waste attitudes or behaviours. The data are presented through a combination of 3D models and 2D textual elements that further elaborate on the 3D models. In this way, the user is engaged by the stereoscopic images and can get more specific information from text that would be difficult to represent in a 3D model. During the development, it was chosen to use models that represent multiple units of food. A first prototype was made and tested in various situations. Later on, models that represent all food waste from one week were implemented along with textual information overlay. The software used for developing the application was Aryzon SDK, Unity and XCode.

During the evaluation, 60% of the survey participants agreed that the app helped with understanding the level of waste. All participants agreed that the app clearly visualises which categories are wasted the most. Notably almost no participants disagreed and no participant strongly disagreed with these statements. Another key finding is that every participant indicated they were surprised by the amount wasted and answered that the waste was more than they expected. When asked what users liked the least, 33% said the textual information. This may be a point to improve upon in further iterations of the app. Furthermore, when directly asked ‘What do you think the mobile application should improve on?’ participants had the opportunity to provide qualitative response. Replies included to provide ‘a more clear presentation of the text, and higher visual qualities of the objects’, to add ‘more of a menu, maybe different restaurants’, to offer a ‘clearer distinction between types of food’, to ‘make the food more 3D shaped’, and finally the ‘Ability to select other weeks/days to see the difference’.

With regards to the immersion, all users felt immersed in the visualisation but all mentioned that the immersion can be improved upon. One suggestion was to increase the polygon count on the 3D models and another suggestion was to use more models in the same art style. The overall experience of the app was rated a 4/5 by the testers. 42% would rather use the application without the headset. Other suggestions for improvements in the application were for a clearer representation of the text.

## Conclusion

To conclude, within the constraints of the limited testing process, the use of an AR application to help reduce food waste is promising. As this research was limited in participants due to the Covid-19 restrictions, no real reduction in food waste could be measured to accompany the study, but this will be considered in future work. However, some suggestions that can be made are as follows. In this case, the most suitable AR platform was a premade SDK (from Aryzon), but in future, this platform could be switched to an online based platform, that gathers data online and updates real time. Additional features could include more realistic models, a greater diversity in products, a menu, and clearer textual information. As for the headset, most users liked reacted positively, but it is strongly suggested to make the app usable both with, and without a headset for practicality. The 3D visualisation of the data was mostly favoured by most users and seems to be a viable representation of the food waste data. Especially when comparing this to more conventional methods such as pie charts or static images.

The research about food waste and AR is still in its infancy and more research needs to be done to be able to draw conclusions on the matter. However, the technology is viable and may contribute to the reduction of food waste in the restaurant business. Further investigation is necessary to expand on the technology and to bring it to consumers and households as well. The current application has limitations with representing the data and intuitiveness. This will need some improvements for larger scale uptake and use in a business setting. Limitations also include the target evaluation group, as the focus of the survey was on one select group of users, who are familiar with smart phone technologies. Other less technologically capable users may provide different responses. In future work, the authors will expand the study to wider user groups to collate more detailed opinions and reflections.

## Supplementary Information

Below is the link to the electronic supplementary material.

Supplementary file1 (DOCX 295 kb)
