# Supplementary material for: Harnessing Augmented Reality for Increasing the Awareness of Food Waste Amongst Dutch Consumers
Source: Augment Hum Res. 2022 Jul 1;7(1):2. doi: 10.1007/s41133-022-00057-7 (PMC9247927; doi:10.1007/s41133-022-00057-7)
Supplement: Supplementary file 1 — Supplementary file1 (DOCX 295 kb) [file 41133_2022_57_MOESM1_ESM.docx]

Appendix A – Questionnaire Format

|  |
| --- |
|  |
|  |
|  |
